# Musculoskeletal pain post-COVID-19 in patients undergoing physical therapy in Saudi Arabia: a cross-sectional study

**DOI:** 10.1186/s12891-023-06647-9

**Published:** 2023-06-21

**Authors:** Ohoud S. Alnamlah, Maha M. Almarwani

**Affiliations:** Rehabilitation Sciences Department, College of Applied Medical Sciences, King Saud University, Riyadh, 11433 Saudi Arabia

**Keywords:** COVID-19, Musculoskeletal pain, Physical activity, Physical therapy

## Abstract

**Background:**

The COVID-19 (coronavirus disease 2019) pandemic has posed a challenge to the physical therapy service. In addition to pandemic-associated treatment interference, many recovered COVID-19 patients developed new or worsening musculoskeletal pain as a sequela of COVID-19, which has been shown to affect the musculoskeletal system. The objective of the study was to examine musculoskeletal pain post-COVID-19 in patients undergoing physical therapy in Saudi Arabia.

**Methods:**

The design of the study was a cross-sectional study. We approached patients attending physical therapy clinics who had COVID-19. Data were collected through an electronic survey consisting of multiple-choice questions related to sociodemographic data and pain. Pain severity was rated on a 10-point numerical rating scale.

**Results:**

A total of 85 recovered COVID-19 patients participated in this study, 30 had musculoskeletal pain prior to getting COVID-19, while 55 acquired it after. The most affected sites for musculoskeletal pain were the lower back and shoulder. Mean pain levels reported increased from 4.48 ± 2.54 pre-COVID-19 to 6.92 ± 8.06 post-COVID-19 (mean difference, 1.61 ± 2.61; t = 5.68; *p* < 0.0001). Mean pain scores did not associate significantly with demographic or clinical factors. Patient responses skewed toward increased pain as well as decreased activity levels after being infected with COVID-19 versus pre-COVID-19 (all *p* < 0.0001).

**Conclusions:**

Recovered COVID-19 patients reported increased pain intensity and frequency, together with reduced activity levels, relative to pre-COVID-19 levels, without effects of sociodemographic or clinical characteristics.

## Background

The coronavirus disease 2019 (COVID-19) pandemic has been a challenging global crisis with far-reaching impacts on healthcare access [1]. The pandemic’s circumstances and pressures have made it difficult in many settings to provide safe, effective, and efficient clinical care, including primary and secondary physiotherapy, in public and private clinics in accordance with the World Health Organization’s (WHO) social distancing and risk-attenuating guidelines [2, 3]. Many patients with non-emergency musculoskeletal symptoms postponed or avoided physiotherapy treatments during the pandemic [4]. Musculoskeletal symptoms, which often include acute or chronic pain in muscles, ligaments, bones, and nerves, represent the most common reason for seeking physical therapy [5].

One of the common manifestations of COVID-19 is musculoskeletal pain. They are less common than the other main symptoms like fever, headache, and cough. But evidence is mounting that COVID-19 can have a significant impact on the nervous and musculoskeletal systems [6, 7]. A recent systematic review of musculoskeletal and neurological features of COVID-19 reported that the prevalence of myalgia and back pain was 19% and 10%, respectively [8]. Sahin et al. found that the most painful body areas in COVID-19-infected people were their limbs and heads. Researchers also reported that the duration of neck and back pain can be longer than expected after the infection has passed [9]. Also, it has been reported that Saudi physicians had higher rates of musculoskeletal disorders after the COVID-19 lockdown. This may be due to a combination of psychological, individual, and work-related factors [10].

Tuzan et al. measured pain intensity using the numeric rating scale (NRS) in 150 post-Covid 19 patients and categorized them into severe and non-severe groups according to American Thoracic Society (ATS) guidelines. NRS scores were almost identical among the two groups. Around 34.0% had at least one comorbidity, with diabetes (23.3%), cardiovascular disease (27.3%), and hypertension (34.0%) [11]. The most frequently reported musculoskeletal symptom was fatigue, followed by myalgia, arthralgia, and back pain. When the authors looked at the location of musculoskeletal pain, back pain was the most frequently reported type. They reported that muscular involvement can be represented as a triangular pattern of myalgia, fatigue, and weakness in COVID-19 and that the main manifestation of myalgia is ischemic myalgia caused by hypoxia [11].

Low physical activity (PA) levels during the quarantine play a role in the prevalence of musculoskeletal pain, such as low back pain and neck pain [12]. This supports the findings of the study, which examined 2044 Italian university students. They compared the physical activity of the students before and after one year of the pandemic and reported a significant reduction in the PA levels and incidence of low back pain (33.5%) and neck pain (43.5%) [13]. The World Health Organization (WHO) recommends limiting sedentary time and performing at least 150 min of PA per week [14].

A recent study investigated the effects of musculoskeletal pain on 90 participants. The pain locations were divided into the following categories: lower limbs, upper limbs, back, head, and neck. Lower limb pain was the most common in COVID-19 patients, followed by head and neck pain. The severity of COVID-19 symptoms, age, general health, smoking, and physical activity levels were all significantly associated with musculoskeletal pain among COVID-19 patients. These factors would be helpful to better understand why some COVID-19 patients have musculoskeletal pain and others do not [15].

These findings highlight the importance and effectiveness of physiotherapy during the COVID-19 crisis. International healthcare is now focused on the needs and treatment of pandemic survivors [16]. It is essential to determine the effects of COVID-19 on the musculoskeletal system and provide multidisciplinary interventions, including physical rehabilitation. To the best of our knowledge, no previous study has examined the musculoskeletal pain and physiotherapy needs of post-COVID-19 patients attending rehabilitation clinics in Saudi Arabia. The purpose of this study was to examine musculoskeletal pain post-COVID-19 in patients undergoing physical therapy in Saudi Arabia.

## Methods

### Design and participants

This was a cross-sectional study that included a convenience sample. The inclusion criteria were recovered COVID-19 patients attending a physical therapy clinic in Saudi Arabia for musculoskeletal pain and aged 20 years or older. This study was approved by the College of Medicine Institutional Review Board at King Saud University (E-21-6506), and all participants provided informed consent prior to participation.

### Sample size

At a significant level of 0.05 and a power of 90%, considering an expected moderate effect size (d = 0.45) [12], the sample size will be at least 85 patients. The sample size calculation was done using G Power ® 3.1.9.4.

### Survey

A self-administered questionnaire in English and Arabic was developed and adapted based on previous studies [11, 13, 15]. Four-part printed and online forms were distributed to recovered COVID-19 patients attending physical therapy clinics. The first part of the questionnaire included questions related to demographic variables. The second part consisted of short descriptive questions related to clinical characteristics, including existing medical conditions, musculoskeletal pain symptoms, and the onset of pain. The third part was a pain severity numerical rating scale (NRS) used to rate one’s musculoskeletal pain before contracting COVID-19 and after recovering from COVID-19. The last part of the questionnaire included Likert-scale questions about pain location, musculoskeletal pain perception, pain relief strategies, physical therapy sessions, and physical activity levels after recovering from COVID-19.

### Outcome measures

Pain intensity using the numeric rating scale (NRS) and Likert scale (5-point scale) scores were used to assess musculoskeletal pain pre- and post-infection with COVID-19 and assess perceptions towards pain and the remedial measures for pain.

### Data collection

Prior to being distributed, the survey was validated in a presurvey of eight respondents to assess the clarity and comprehensibility of the questions, face validity, and content validity. Content validity was adjusted based on feedback from physiotherapists who participated in the presurvey. Face validity was adjusted by modifying choices to better fit the questions. The questions were adjusted based on their answers and their recommendations.

Patients attending rehabilitation clinics in Saudi Arabia were invited to participate by phone and email. Their contact information was extracted from electronic medical records. Participants were able to fill out online questionnaires or in person. The questionnaires were prefaced with information summarizing the purpose of the study.

### Statistical analysis

All statistical analyses were performed using SPSS version 27.0. We computed appropriate descriptive statistics to describe the study sample. A nonparametric Pearson’s chi-square fitness test was used to observe the statistical significance of the observed categorical responses to different items in each of the perception and remedial measures items. Paired t-test to compare the mean pain scores between before and after the COVID-19 infection. Also, the student’s t-test for independent samples and one-way ANOVA were used to compare the mean pain score difference in relation to the categorical variables, which have two or more categories. A *p*-value of 0.05 was used to report the statistical significance of the results.

## Results

The study sample included 85 subjects, including 59 women (69.4%) and 26 men (30.6%). Their ages were distributed as follows: 20–30 years, 19 (22.4%); 31–40 years, 22 (25.9%); 41–50 years, 20 (23.5%); 51–60 years, 15 (17.6%); >60 years, 9 (10.6%). The majority of the participants were married (n = 50; 58.8%) (Table 1).

**Table 1:** Characteristics of study subjects

Characteristics	N (%)
***Age (years)***	
20-30	19 (22.4)
31–40	22 (25.9)
41–50	20 (23.5)
51–60	15 (17.6)
> 60	9 (10.6)
***Gender***	
Male	26 (30.6)
Female	59 (69.4)
***Educational level***	
High school	19 (22.3)
Bachelor	53 (62.4)
Master	9 (10.6)
PhD	4 (4.7)
***Marital Status***	
Single	24 (28.2)
Married	50 (58.8)
Divorced	7 (8.2)
Widow	4 (4.7)

*Note*: Data are presented as frequency and percentage

Regarding the medical histories of the participants, the majority (55.3%) indicated that they had no pre-existing diseases. However, the remaining participants had one disease. Regarding time since COVID-19 infection, most of the 28 participants (32.9%) were infected more than a year prior. Thirty of the participants (35.3%) indicated that they had experienced musculoskeletal pain prior to getting COVID-19, while 55 (64.7%) indicated that their pain started after they were infected. Regarding pain location, the most painful body parts affected were the lower back (58.8%), shoulder (38.1%), and knee (27.4%) respectively (Table 2).

**Table 2:** Clinical characteristics of study subjects

Characteristics	N (%)
***Medical condition***	
Diabetes	11 (12.9)
Hypertension	6 (7.1)
Hyperlipidaemia	6 (7.1)
Other	15 (17.6)
Without any medical condition	47 (55.3)
***Time of having a COVID-19 infection***	
Less than a month	2 (2.4)
1 to 3 months	20 (23.5)
4 to 6 months	15 (17.6)
7 to 12 months	20 (23.5)
> 1 year	28 (32.9)
***Onset of musculoskeletal pain***	
Before getting the COVID-19 infection	30 (35.30)
After getting the COVID-19 infection	55 (64.70)
***Location of musculoskeletal pain***	
Neck	20 (23.8)
Shoulder	32 (38.1)
Elbows	6 (7.1)
Wrists/Hands	10 (11.9)
Lower back	50 (58.8)
Hips/Thighs	18 (21.4)
Knees	23 (27.4)
Ankles/Feet	18 (21.4)

*Note*: Data are presented as frequency and percentage

The study exhibited a highly significant increase in overall mean (SD) NRS pain scores. The comparison study subjects’ mean scores of pain between before and after being infected with COVID-19, in which the mean difference of pain scores is highly statistically significant, show that the mean pain score is statistically significantly increased post-COVID-19 (6.92) when compared with the mean pain score pre-COVID-19 (4.48). The difference in the mean pain score (1.61) is highly statistically significant (t = 5.68, *p* < 0.0001) (Table 3).

**Table 3:** Musculoskeletal pain pre- and post-COVID-19

Stage of pain	Pain score	Difference	*t* Value	*p* Value
Musculoskeletal pain pre-COVID-19	4.48 (2.54)	1.61(2.61)	5.68	< 0.0001*
Musculoskeletal pain post-COVID-19	6.92 (8.06)			

Note: Values are mean (standard deviation) unless otherwise noted. * A significant difference (p < 0.05) on pain score pre vs. post COVID-19 using paired t-tests

The comparison of the mean pain score difference (before and after being infected with COVID-19) in relation to the socio-demographic and clinical characteristics of study subjects shows no statistically significant difference in the mean pain score difference in relation to the socio-demographic and clinical characteristics (Table 4).

**Table 4:** Musculoskeletal pain pre- and post-COVID-19 in relation to the socio-demographic characteristics of study subjects

Characteristics	Difference of pain score	F-value/t-value	*p* value
***Age (years)***			
20–30	1.53 (2.75)	1.44	0.229
31–40	2.59 (2.67)		
41–50	0.75 (2.63)		
51–60	1.27 (2.40)		
> 60	1.89 (2.15)		
***Gender***			
Male	1.77 (2.32)	0.367	0.715
Female	1.54 (2.75)		
***Marital Status***			
Single	2.33 (2.37)	1.09	0.356
Married	1.32 (2.85)		
Divorced	0.86 (0.90)		
Widow	2.25 (2.63)		
***Onset of pain in musculoskeletal system***			
Before getting the COVID-19 infection	0.93 (2.15)	-1.79	0.077
After getting the COVID-19 infection	1.98 (2.78)		
***Medical condition***			
Diabetes	2.09 (2.81)	2.053	0.095
Hypertension	1.17 (2.14)		
Hyperlipidaemia	1.17 (2.40)		
Other	1.73 (2.05)		
Without any medical condition	1.87 (2.68)		
***Time of having the COVID-19 infection***			
Less than a month	4.00 (0.00)	0.963	0.433
1 to 3 months	1.05 (2.58)		
4 to 6 months	2.20 (2.34)		
7 to 12 months	1.25 (2.94)		
> 1 year	1.79 (2.59)		

Note: Values are mean (standard deviation) unless otherwise noted. *A significant difference (p < 0.05) on pain score pre vs. post COVID-19 using independent sample t-tests and one way ANOVA

The comparison of study subjects’ perceptions of pain in the musculoskeletal system after being infected with COVID-19 is given in (Table 5). For the perception item, “After getting the COVID-19 infection, did the pain in the musculoskeletal system increase? The subjects have responded as strongly agree by 17.6%, as agree by 38.8%, 20% of them as neutral and remaining as disagree and strongly disagree (*p* < 0.0001). For the perception item, after getting the COVID-19 infection, did the frequency of pain increase? The study subjects responded strongly agree by 9.4%, agree by 43.5%, 23.5% of them as neutral and remaining as disagree and strongly disagree (p < 0.0001).

The comparison of study subjects’ perceptions towards the remedial measures for pain in the musculoskeletal system after being infected with COVID-19 was carried out using 5 perception statements. The distribution of these responses indicates statistical significance (*p* < 0.0001). For the statement, after getting the COVID-19 infection, did the number of physical therapy sessions increase? 30.6% of them were neutral, 29.4% agreed, and 37.7% disagreed or strongly disagreed.

There is highly statistically significant difference in the distribution of these proportions (p < 0.0001). For the other 3 perception statements: “After getting the COVID-19 infection, did the number of physical exercises decrease?” “After getting the COVID-19 infection, did the amount of time for physical activity reduce?” and “After getting the COVID-19 infection, did the number of hours that you spent sitting during the day increase? 36.5%, 35.3% and 35.3% of them were neutral, 35.3%, 43.5% and 47.0% of them agreed or strongly agreed with these three statements (Table 5).

**Table 5:** Study subjects’ perceptions towards musculoskeletal pain post-COVID-19

Perception items	N (%)	X^2^-value	p-value
***Musculoskeletal pain increases post COVID-19***			
Strongly disagree	6 (7.1)	22.94	< 0.0001*
Disagree	14 (16.5)		
Neutral	17 (20.0)		
Agree	33 (38.8)		
Strongly agree	15 (17.6)		
***The frequency of musculoskeletal pain increases post COVID-19***			
Strongly disagree	4 (4.7)	38.82	< 0.0001*
Disagree	16 (18.8)		
Neutral	20 (23.5)		
Agree	37 (43.5)		
Strongly agree	8 (9.4)		
***Type of plan for pain relief***			
Do exercises	28 (32.9)	27.23	< 0.0001*
Take a pain reliever	37 (43.5)		
Sleep	14 (16.5)		
Other	6 (7.1)		
***The number of physical therapy sessions increased post-COVID-19***			
Strongly disagree	14 (16.5)	22.35	< 0.0001*
Disagree	18 (21.2)		
Neutral	26 (30.6)		
Agree	25 (29.4)		
Strongly agree	2 (2.4)		
***The number of physical exercises decreased post-COVID-19***			
Strongly disagree	5 (5.9)	34.94	< 0.0001*
Disagree	19 (22.4)		
Neutral	31 (36.5)		
Agree	26 (30.4)		
Strongly agree	4 (4.7)		
***The amount of time for physical activity has reduced post-COVID-19***			
Strongly disagree	3 (3.5)	30.21	< 0.0001*
Disagree	15 (17.6)		
Neutral	30 (35.3)		
Agree	29 (34.1)		
Strongly agree	8 (9.4)		
***The number of hours that you spend sitting during the day increased post-COVID-19***			
Strongly disagree	1 (1.2)	43.53	< 0.0001*
Disagree	14 (16.5)		
Neutral	30 (35.3)		
Agree	32 (37.6)		
Strongly agree	8 (9.4)		

Note: N: number, %: percentage, X^2^-value: related to Pearson’s Chi-square test, * significance level was set at *p* < 0.05

## Discussion

The present study showed that recovered COVID-19 patients attending physical therapy clinics in Saudi Arabia experienced increased pain intensity and frequency after being infected with COVID-19, relative to their pre-illness levels. Additionally, based on survey self-reporting, we found that our recovered COVID-19 patients sample tended to exercise less, spend less time active, and spend more time sedentary compared to their behavior prior to contracting COVID-19. While slightly less than a third of the respondents engaged in exercises as part of their pain remediation plans, a substantially larger proportion, 43.5%, relied on pain medication use, either alone or with other strategies.

The findings that NRS pain scores increased by about 2.5 points on average after recovery from COVID-19 versus levels prior to the illness are clinically concerning. When asked whether their pain had increased in intensity and frequency, the answers were skewed toward agree or strongly agree and away from disagree or strongly disagree, suggesting there was a non-random shift toward more severe and more frequent pain before versus after their being ill with COVID-19. The pain worsening and persistence in recovered COVID-19 patients are consistent with prior studies reporting similar phenomena in other recovered COVID-19 patient populations [8, 9, 11, 15]. In the case series conducted by Carfi et al., one-quarter to one-half of recovered patients with COVID-19 have been reported with a clinical presentation and symptoms that include myalgias, generalized weakness, and arthralgia as persistent symptoms following COVID-19 infection [17]. Similarly, Alkodaymi et al. (2022) investigated the prevalence of persistent symptoms 12 weeks post COVID-19 infection among the 257,348 population after screening 3209 studies and found about 22% had myalgia [18].

In our study, differences in mean pain scores did not differ significantly among age groups, gender groups, marital status groups, preexisting condition groups, or time since infection groups. There is a significant trend suggesting that those whose pain emerged after contracting COVID-19 might experience a greater difference (worsening) in pain than those whose pain onset preceded their infection with COVID-19.

With respect to the localization of musculoskeletal pain, the finding that low back pain was prevalent in recovered COVID-19 patients agrees with the study by Sagat et al. They found an increase in the severity of low back pain during quarantine, particularly between the ages of 35 and 49. These results were supported by the following explanations: telecommuting and long work hours, lower physical activity and high-stress levels [12]. However, Tuzun et al. found that the most common pain presentation in recovered COVID-19 patients was widespread pain, followed by back pain [11]. Multiple risk factors were found to be significantly associated with musculoskeletal pain in sample of recovered COVID-19 patients, including the severity of COVID-19 symptoms, older age, poor general health, smoking, and a low physical activity level [15]. This is an agreement with our study, majority reported decreased physical activity levels. Regarding the general health, 38 of the participants had medical condition and the remaining 47 were medically fit.

Low physical activity may be a risk factor for pain worsening [12, 19]. The present findings of reduced activity levels across assessment items (physical exercise, time spent active, and time spent sedentary) suggest that healthcare providers should work with recovered COVID-19 patients suffering from persistent musculoskeletal symptoms to improve their activity levels. The findings of a large meta-analysis of reviews suggested that physical activity can reduce pain, improve physical function, and thus improve quality of life [20]. Exercise has direct and indirect benefits for patients with chronic pain. Another recent systematic review confirmed that the implementation of exercise regimens can enable patients to reconceptualize pain in a manner that can lead to the gradual reversal of the so-called vicious cycle of pain [21].

Regarding the mechanism by which COVID-19 leads to musculoskeletal symptoms, there are several, not mutually exclusive, possibilities. Firstly, lactate dehydrogenase (LDH) and creatine kinase, which are released in response to tissue damage, have been reported to be elevated in COVID-19 patients with musculoskeletal symptoms [22]. Recovered COVID-19 patients with fatigue have been reported to have significantly higher LDH levels than recovered COVID-19 patients without fatigue [8]. A second possible explanation is that musculoskeletal pain is a sequela of a COVID-19 inflammatory cytokine storm, wherein a burst of pro-inflammatory cytokines, such as interleukin-6, is produced [23, 24] Thirdly, muscular pain, in at least some cases, may reflect poor vascularization of muscles related to COVID-19 associated thromboses [25, 26]. Finally, coronavirus spike proteins, which mediate the establishment of COVID-19, interact with ACE2 (angiotensin-converting enzyme 2) in muscles. However, the significance of this relationship for the course and symptoms of COVID-19 is not yet known [27, 28].

There are some limitations that should be addressed in interpreting our results. Firstly, this was a convenience sample which may not have represented the entire Saudi population. Secondly, response bias to the self-administrated questionnaire may confound results. Thirdly, the reliability and validity of the self-administrated questionnaire have not been investigated. However, the questionnaire was adapted from previous studies. Lastly, it is important to note that musculoskeletal pains are multifactorial with many confounding variables that could not be controlled in this study including psychological, physical and social factors. Future studies with a larger sample size and sufficient statistical power might be needed to identify this association in the multivariate analysis.

## Conclusion

Recovered COVID-19 patients in Saudi Arabia are experiencing increased pain intensity and frequency relative to prior to their illness. Similar changes were observed across age, gender, marital status, and education levels, as well as across comorbidity and time since COVID-19 infection. While slightly less than a third of recovered COVID-19 patients in this study being treated for musculoskeletal pain became less active relative to their pre-illness activity levels. Physical therapists should pay more attention to recovered COVID-19 patients suffering from persistent musculoskeletal symptoms by providing the required exercises and improving their activity levels.

## Data Availability

The data that support the findings of this study are available upon request to the correspondent author.

## List of abbreviations

ATS: American Thoracic Society
COVID-19: The coronavirus disease 2019
LDH: Lactate dehydrogenase
NRS: Numeric rating scale
PA: Physical activity
SPTA: Saudi Physical Therapy Association
WHO: World Health Organization
